# Mitochondrial pyruvate carrier 1 expression controls cancer epithelial‐mesenchymal transition and radioresistance

**DOI:** 10.1111/cas.13980

**Published:** 2019-04-04

**Authors:** Yuji Takaoka, Masamitsu Konno, Jun Koseki, Hugh Colvin, Ayumu Asai, Keisuke Tamari, Taroh Satoh, Masaki Mori, Yuichiro Doki, Kazuhiko Ogawa, Hideshi Ishii

**Affiliations:** ^1^ Department of Radiation Oncology Osaka University Suita Japan; ^2^ Department of Medical Data Science Osaka University Suita Japan; ^3^ Department of Frontier Science for Cancer and Chemotherapy Osaka University Osaka Japan; ^4^ Department of Gastroenterological Surgery Osaka University Suita Japan; ^5^ Department of Surgery and Science Graduate School of Medical Sciences Kyushu University Fukuoka Japan

**Keywords:** EMT, glutamine, metabolism, mitochondria pyruvate career, radiation therapy

## Abstract

Mitochondrial pyruvate carrier (MPC) is known to cause different expressions in normal and cancer cells. We observed a change in phenotype with the suppression of MPC expression. We knocked down MPC1 and/or MPC2 using siRNA or shRNA. We observed its cell morphology and accompanying molecular marker. Furthermore, the radioresistance of the MPC knockdown cell line was examined using a colony formation assay. MPC1‐suppressed cells changed their morphology to a spindle shape. Epithelial‐mesenchymal transition (EMT) was suspected, and examination of the EMT marker by PCR showed a decrease in E‐cadherin and an increase in fibronectin. Focusing on glutamine metabolism as the mechanism of this phenomenon, we knocked down the glutamine‐metabolizing enzyme glutaminase (GLS). EMT was also observed in GLS‐suppressed cells. Furthermore, when MPC1‐suppressed cells were cultured in a glutamine‐deficient medium, changes in EMT markers were suppressed. In addition, MPC1‐suppressed cells also increased with a significant difference in radioresistance. Decreased MPC1 expression favorably affects EMT and radioresistance of cancer.

**Abbreviations**
CRC: colorectal cancer
d‐2HG: d‐2‐hydroxyglutarate
EMT: epithelial‐mesenchymal transition
GLS: glutaminase
GSH: glutathione
HIF‐1: hypoxia‐inducible factor‐1
MPC: mitochondrial pyruvate carrier
OCR: oxygen consumption rate
PPP: pentose phosphate pathway
TCA: tricarboxylic acid

## 1 INTRODUCTION

In cancer cells, glycolysis, which is the anaerobic ATP production pathway, is enhanced. Therefore, pyruvate uptake into the mitochondria is decreased. This metabolic change is well known as the Warburg effect.^1^ It is believed to be an adaptation to hypoxia in the tumor.

Glucose is metabolized to pyruvate in the glycolysis system. Pyruvate uptake into the mitochondria is metabolized by the TCA cycle and eventually used for aerobic ATP production through oxidative phosphorylation. Glycolytic anaerobic ATP production produces 2 ATP per glucose molecule, whereas aerobic ATP production produces 36 ATP per glucose molecule. Therefore, glycolytic anaerobic ATP production is inefficient. Glucose‐6‐phosphate, an intermediate product of glycolysis, is the starting point of PPP, which is involved in the production of nucleic acid synthesis and reduced GSH, which is a major radical scavenger. Today, as one of the hypotheses, the Warburg effect is advantageous for the supply of bioproducts necessary for cancer cell survival and proliferation.^2^

Recent studies have suggested that understanding cancer glucose metabolism is very important in cancer treatment. As part of it, HIF‐1 is considered to cause hyperexpression in the hypoxic region of cancer, but the HIF‐1α control glycolysis system increases metabolite flux to PPP. Increased flux to PPP has been reported to affect radioresistance and gemcitabine (a nucleic acid antimetabolite) tolerance.^3^, ^4^ Radiation therapy, chemotherapy, and chemoradiation therapy as well as surgery are the most fundamental treatments in today's cancer treatment.

Mitochondrial pyruvate carrier is present in the inner membrane of the mitochondria and is a membrane protein that takes up pyruvate into the mitochondria. In studies using yeast, MPC contains subunits of MPC1, MPC2, and MPC3 and exists as a multicomplex.^5^ In humans, reports mentioning MPC3 are very few, but a deficiency of MPC expression has been shown to be involved in the onset of familial metabolic disorders.

Recent reports have indicated that MPC1 often shows low expression in many carcinomas compared to normal cells and MPC2 tends to be highly expressed.^6^ It is also known that low MPC1 expression in colon cancer, renal cell carcinoma, and lung cancer has poor survival.^6^ In contrast, the effects of changes in the expression of the MPC subtype on the tumor are still poorly understood. Previously, Ohashi et al^7^ reported that EMT was induced in the cholangiocellular carcinoma cell line by suppression of MPC1 expression. In a developmental study, we investigated the possibility that EMT as a result of the suppression of MPC expression is caused by a compensatory mitochondrial metabolism by glutamine and MPC related to resistance to radiation therapy. Regarding cancer cell metastasis and survival when receiving treatment, we found that MPC produces a suitable metabolic environment.

## 2 MATERIALS AND METHODS

### 2.1 Comparison of MPC1 expressions for the same patients

We analyzed the differences of MPC1 expression level between normal and tumor tissues in the same patients with GSE89393 and GSE16515 from the Gene Expression Omnibus (GEO) in NCBI. GSE89393 is expression profiling data by high throughput sequencing for colon cancer cases, which included the expression data of six patients with absence of a tumor case. We selected five patients with the presence of expression data of paired normal and tumor tissues for the analysis of colon cancer. GSE16515 contains microarray data of paired tumor and normal tissues from 16 cases of pancreatic cancer, which were subjected to the present study.

### 2.2 Cell line and culture

Human pancreatic cancer cell lines (MIA PaCa‐2 and PANC‐1) and CRC cell lines (HT29, HCT116, CaCo‐2, LoVo, CaR1, RKO, and SW480) were used. Cells were cultured in DMEM (1.0 g/L glucose with l‐glutamine and sodium pyruvate, liquid 08456‐36; Nacalai Tesque, Inc., Kyoto, Japan) with 10% FBS and 1% penicillin‐streptomycin mixed solution. Cells were incubated in an incubator with 5% CO_2_.

### 2.3 Transfection of vector for knockdown of any genes

Small interfering RNAs were purchased from Abgent (San Diego, CA, USA). siRNAs were transfected using Lipofectamine 3000 reagent (Thermo Fisher Scientific, Waltham, MA, USA) according to the manufacturer's protocol.

Short hairpin RNAs were purchased from Abgent. shRNAs were transfected using Lipofectamine 3000 reagent and P3000 reagent (Thermo Fisher Scientific) according to the manufacturer's protocol. Transfected cells were selected with puromycin.

### 2.4 Quantitative real‐time polymerase chain reaction

Total RNAs were extracted from cells using Isogen (Nippon Gene, Tokyo, Japan), and complementary DNAs were synthesized from total RNAs using ReverTra Ace qPCR RT Master Mix (Toyobo, Osaka, Japan). Real‐time PCR was carried out using THUNDERBIRD SYBR qPCR Mix (Toyobo) and LightCycler (Roche, Basel, Switzerland). Primers are as follows: β‐actin, forward primer 5′‐agagctacgagctgctgac‐3′ and reverseprimer 5′‐acactgtgttggcgtacag‐3′; MPC1, forward primer 5′‐caatggaaaaggaagaacaagg‐3′ and reverse primer 5′‐aggcagcagagagttggtttag‐3′;MPC2, forward primer 5′‐agtcagatgagtcagcaaagca‐3′ and reverseprimer 5′‐agaacctcttcattgtggccta‐3′; E‐cadherin, forward primer5′‐acaccatcctcagccaaga‐3′ and reverse primer 5′‐cgtagggaaactctctcggt‐3′;fibronectin, forward primer 5′‐ctggccgaaaatacattgtaaa‐3′ and reverseprimer 5′‐ccacagtcgggtcaggag‐3′; Slug, forward primer 5′‐tcagctcaggagcatacagc‐3′ and reverse primer 5′‐gactcactcgccccaaaga‐3′; and vimentin, forward primer 5′‐tccagcagcttcctgtaggt‐3′ and reverse primer 5′‐ccctcacctgtgaagtggat‐3′.

### 2.5 Western blotting analysis

Whole‐cell lysates including total protein were prepared using RIPA buffer (Thermo Fisher Scientific). Electrophoresis was transferred to the membrane using a semidry system. The membranes were incubated at 4°C overnight with E‐cadherin antibody (Cell Signaling Technology, Danvers, MA, USA), GLS antibody (Abgent), and ACTB antibody followed by 1‐hour incubation with HRP‐linked antirabbit IgG (GE Healthcare Biosciences, Piscataway, NJ, USA; 1:100 000 dilution) at room temperature. The antigen‐antibody complex was detected with an ECL Prime Western Blotting Detection Kit (GE Healthcare Biosciences).

### 2.6 Wound healing assay

To confirm the migration ability, a wound healing assay was carried out. Cells were seeded into a six‐well plate and allowed to grow from 90% to 100% confluence in DMEM, and cell monolayers were then wounded by a 200‐mL pipette tip.^8^ The distance covered by migrated cells was quantified at 0, 3, 6, 12, and 24 hours.

### 2.7 Flux analysis

In the present study, flux analysis was carried out using an extracellular flux analyzer XFe24 (Agilent Technologies, Tokyo, Japan). XFe24 measures the extracellular OCR and pH change using photoluminescence. Respiratory status was calculated by Wave software version 2.6.0.31 (Primetech Corp., Osaka, Japan).

#### 2.7.1 Mito stress test

Basic respiratory status (ATP production, anaerobic respiration [glycolysis], and aerobic respiration [TCA cycle]) was assessed by Mito Stress Test Kit (Primetech Corp.) according to the manufacturer's protocol.

#### 2.7.2 Mito fuel flex test

Capacity and dependence on glutamine in ATP production were assessed using the Mito Fuel Flex Test Kit (Primetech Corp.) according to the manufacturer's protocol.

### 2.8 Colony formation assay

Colony forming ability was assessed using a colony formation assay. Cells were seeded into 6‐cm dishes and irradiated at 0, 4, and 8 Gy on the day after seeding. Fourteen days later, the number of colonies was counted.

### 2.9 Statistical analyses

Data were analyzed using Excel or JMP. Statistical significance of obtained data was determined using Student's *t* test. *P*‐value <0.05, <0.01 is shown in figures by *, **, respectively.

## 3 RESULTS

### 3.1 Effects of low MPC expression on the EMT phenomenon

**Figure 1:**
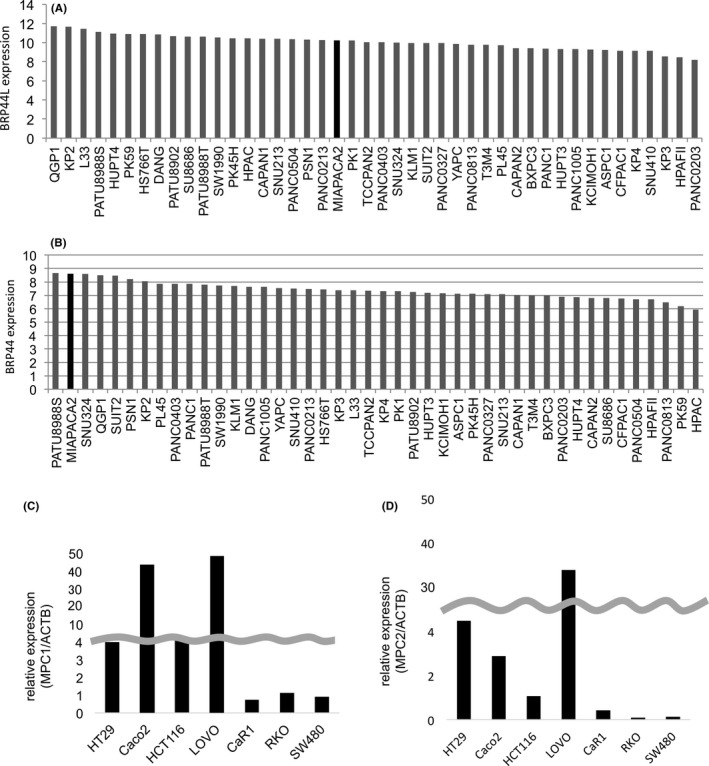
Mitochondrial pyruvate carrier 1/2 (MPC1/2) expression in human pancreatic cancer and colorectal cancer (CRC) cell lines. A,B, Relative MPC1 and MPC2 expression in The Cancer Genome Atlas (TCGA) database. C,D, MPC1 and MPC2 expression in seven CRC cell lines in real‐time PCR

We studied the expression level of MPC1 using the NCBI GEO dataset. These data showed that the expression level of MPC1 was downregulated in colon and pancreatic cancer tissues compared with normal tissues in colon and pancreas (Figure S1A,B). Then we studied the significance of the downregulation for MPC1 expression. To assess the function of MPC for cancer malignancy, we surveyed MPC expression in pancreatic cancer and CRC cell lines. In pancreatic cancer, we used The Cancer Genome Atlas (TCGA) database (Figure 1A,B). BRP44 and BRP44L are gene names coding MPC2 and MPC1, respectively. There was no cell line with more than a twofold expression change, and it seemed that there was no significant difference in expression in the pancreatic cancer cell line. Furthermore, we examined MPC expression in seven CRC cell lines using real‐time PCR (Figure 1C,D), and we conducted subsequent experiments using MIA PaCa‐2 and HT29 as targets for MPC knockdown.

**Figure 2:**
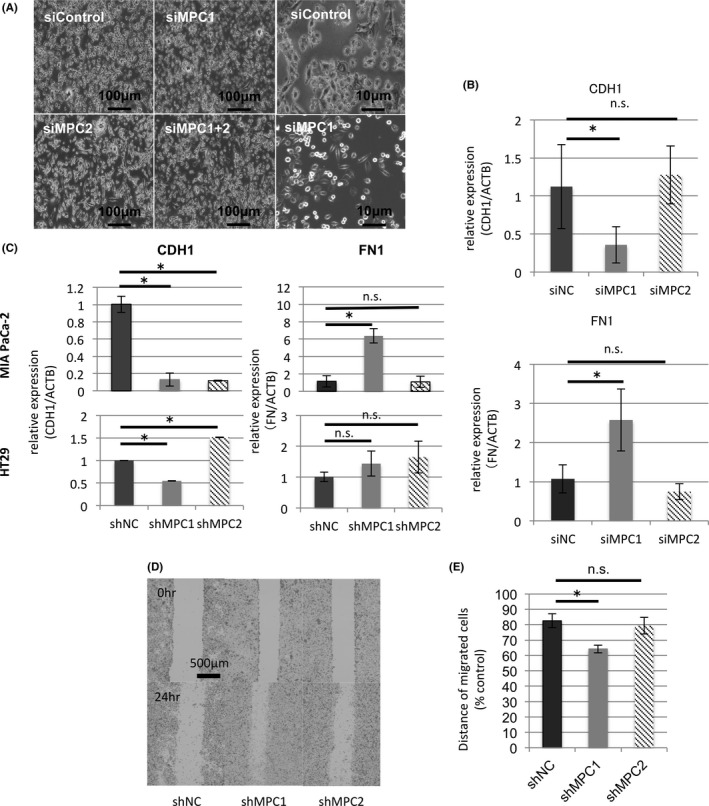
Effects of low mitochondrial pyruvate carrier (MPC) expression on the epithelial‐mesenchymal transition phenomenon. A, Cell shapes in siMPC MIA PaCa‐2. Right, characteristic forms: round and spindle shapes. B,C, Relative E‐cadherin and fibronectin expression in real‐time PCR. D, Wound healing assay in shMPC MIA PaCa‐2. Images were acquired at 0 and 24 h. E, Intercellular distance at 24 h normalized with 0 h as 100%. Data are mean ± SD (n = 3). *P < 0.05; n.s., nonsignificant

To investigate the effect of suppression of MPC expression, we tried to knock down MPC using siRNA (Figure S2). We found that the knockdown resulted in a change of cell shape to a spindle‐like one, compared with siControl cells (Figure 2A). In our previous report, EMT changes as a result of inhibition of MPC expression have been reported in intrahepatic cholangiocarcinoma.^7^ We evaluated the EMT marker using real‐time PCR to determine whether this change was caused by EMT using MIA PaCa‐2. Although there was no obvious change in MPC2 knockdown, MPC1 knockdown using siRNAs or shRNAs showed a decrease in E‐cadherin and an increase in fibronectin (Figure 2B,C). To determine whether this change was applicable to other cells, we also confirmed the EMT marker in the colon cancer cell line HT29. In HT29, the level of E‐cadherin declined in the MPC1 knockdown group. Although the change was not dominant in fibronectin, it showed an upward trend of expression (Figure 2C). To investigate the migratory ability of these cells, we investigated the migration distance of the cells using a wound healing assay. In migration distance, shMPC1 cells also showed a significant increase in migration ability (Figure 2D,E). These results suggest that the suppression of MPC1 expression is involved in the control of CDH1 expression in cancer cells.

### 3.2 Glutaminase expression induces EMT

Next, we considered the mechanism wherein MPC knockdown induces EMT. Therefore, we focused on glutamine metabolism. It is known that the replenishment reaction of the TCA cycle by the glutamine pathway is carried out when MPC is inhibited.^9^ Recent reports have suggested that D‐2HG, which is known as an oncometabolite, may be elevated in glutamine metabolites in colon cancer cells that do not have IDH mutation, further suggesting that D‐2HG is involved in EMT.^10^ We surmised that MPC knockdown decreased the supply of pyruvic acid to the TCA cycle and that the glutamine supply and metabolism might have been enhanced as a compensatory replenishment reaction. Therefore, we first examined whether glutamine metabolism in the mitochondria induces EMT.

**Figure 3:**
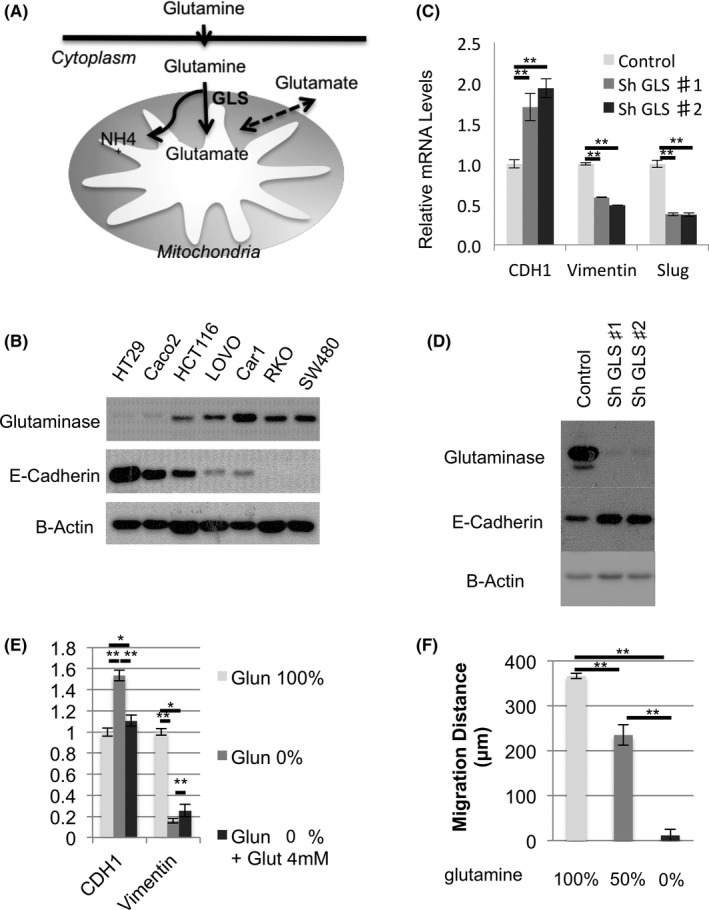
Glutaminase (GLS) expression induces epithelial‐mesenchymal transition (EMT). A, Glutamine metabolism in the mitochondria. B, Western blot analysis of GLS and E‐cadherin in seven colorectal cancer cell lines. C, Relative expression of CaR1 EMT markers (CDH1, vimentin, and Slug) in real‐time PCR. D, Western blot analysis of E‐cadherin expression in shGLS CaR1. E, Relative expression of EMT markers (CDH1 and vimentin) in glutamine (Glun)‐deprived DMEM with or without 4 mmol/L glutamate (Glut). Measurement was carried out 48 h after seeding. F, Wound healing assay of CaR1 with glutamine (4, 2, and 0 mmol/L). Data are mean ± SD (n = 3). *P < 0.05; **P < 0.01; n.s., nonsignificant

To investigate whether glutamine induces EMT, GLS expression and E‐cadherin expression, which metabolize glutamine to glutamate and NH3, were investigated (Figure 3A). Surprisingly, there was a tendency for GLS and E‐cadherin to be inversely correlated (Figure 3B). To investigate the influence on EMT using GLS knockdown cell line, we chose CaR1, which is the highest GLS expressed cell line. E‐cadherin expression was elevated in CaR1‐suppressed GLS, and vimentin and Slug expression were decreased (Figure 3C,D). We considered the possibility that the metabolism of glutamine to glutamate by GLS plays an important role in EMT. In cells cultured in a glutamine‐deficient medium, the increase in E‐cadherin expression and the decrease in vimentin expression were observed as similar to the suppression of GLS expression. In the DMEM plus 4 mmol/L glutamate group, E‐cadherin expression was greatly reduced and became comparable to the glutamine‐present medium group (Figure 3E). Migration also showed a remarkable decrease in migration distance in the glutamine‐deficient medium (Figure 3F).

It has been suggested that glutamine metabolism by GLS is involved in the control of EMT. According to our experimental results, the metabolite after glutamate is involved in the transcriptional regulation of EMT.

### 3.3 Mitochondrial pyruvate carrier knockdown upregulates GLS

To ascertain whether EMT with MPC suppression is the same as GLS‐induced EMT, we investigated whether MPC suppression affects glutamine metabolism.

**Figure 4:**
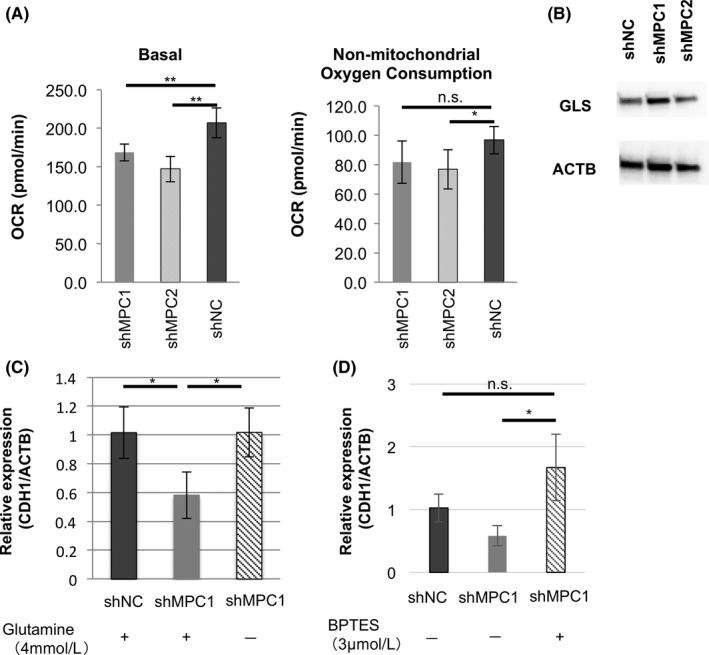
Mitochondrial pyruvate carrier (MPC) knockdown upregulates glutaminase (GLS). A, Oxygen consumption rate (OCR) in shMPC MIA PaCa‐2. Left, basal respiration; right, nonmitochondrial oxygen consumption. Analysis was carried out using flux analyzer XFe24 and wave software. B, Western blot analysis of GLS expression in shMPC MIA PaCa‐2. C, Relative CDH1 expression in shMPC MIA PaCa‐2 with or without 4 mmol/L glutamine. D, Relative CDH1 expression in shMPC MIA PaCa‐2 with or without 3 μmol/L BPTES. Data are mean ± SD (n = 3). *P < 0.05; **P < 0.01; n.s., nonsignificant

We first confirmed the respiratory state of the cells with a flux analyzer using the MIA PaCa‐2 shMPC cell line. The flux analyzer XFe24 measured OCR as an index of cell breathing. The basal respiratory state showed a significant decrease compared to the negative control group in the MPC‐suppressed group (Figure 4A). This reduction rate was considered to be consistent with a recent report that used UK‐5099, which is an MPC inhibitor.^11^, ^12^ In contrast, nonmitochondrial oxygen consumption tended to decrease under MPC1 suppression but not to a significant level (Figure 4A). Although there is no clear basis for the decrease, it may be caused by adaptation to the use of other energy substrates such as glutamine and fatty acids.

Next, to investigate whether glutamine metabolism is enhanced by the suppression of MPC expression, GLS expression was examined by western blotting. Interestingly, the suppression of MPC1 expression alone enhanced GLS expression (Figure 4B). In addition, cells were cultured in a glutamine‐deficient medium under the suppression of MPC1 expression. E‐cadherin recovered its expression to almost the same level as the negative control (Figure 4C). Furthermore, similar results were obtained in the experiment of cell culture in the medium containing BPTES, a glutaminase inhibitor (Figure 4D). In contrast, in the analysis using the flux analyzer, the MPC1 cell line showed no significant enhancement in glutamine dependence and capacity (Figure S3A,B). In summary, GLS expression was enhanced only under the suppression of MPC1 expression, suggesting an increase in glutamine metabolism. The change of E‐cadherin was reset under glutamine deficiency. It seemed likely that MPC expression was similar to EMT derived from glutamine metabolism.

### 3.4 With low MPC expression, cell lines acquire radioresistance

**Figure 5:**
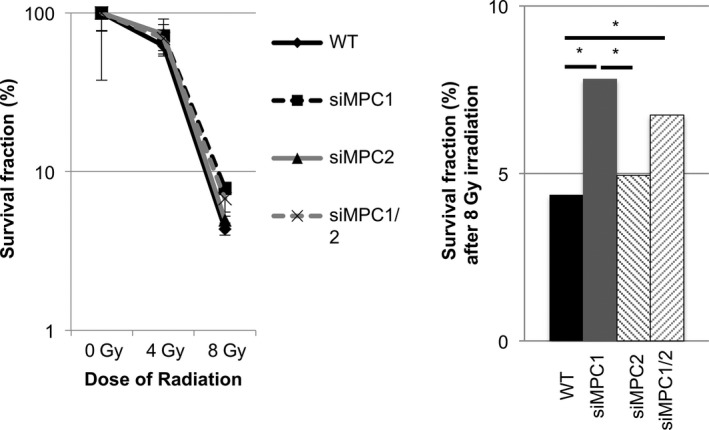
With low mitochondrial pyruvate carrier (MPC) expression, cell lines acquire radioresistance. A, Survival fraction of colony using MIA PaCa‐2 each irradiated at 0, 4, and 8 Gy. B, Survival fraction after irradiation at 8 Gy. Data are mean ± SD (n = 3). *P < 0.05

Finally, in addition to EMT induced by cancer metabolism, we were interested in the possibility that the decrease in MPC expression would enhance radioresistance. To investigate whether MPC expression is involved in radioresistance, irradiation was carried out on cells that knocked down MPC (Figure S2). Twenty‐four hours after cell seeding, single X‐ray irradiation by ^137^Cs was done followed by culturing for 2 weeks. After 2 weeks, the number of colonies grown in each well was counted, and the survival rate of cells maintaining colony forming ability was calculated. Surprisingly, again, a significant survival improvement was confirmed only in cell lines that knocked down MPC1 (Figure 5A,B). This was also observed with permanent suppression of MPC expression using shRNA (Figure S4).

In summary, it was shown that decreased MPC1 expression in cancer cells contributed not only to the metastatic potential of the cells but also to the acquisition of resistance to radiation therapy and was involved in improving the cancer survival rate.

## 4 DISCUSSION

In the present study, we showed that suppression of MPC1 expression leads to EMT in pancreatic cancer and CRC cell lines as well as contributes to the acquisition of radioresistance.

**Figure 6:**
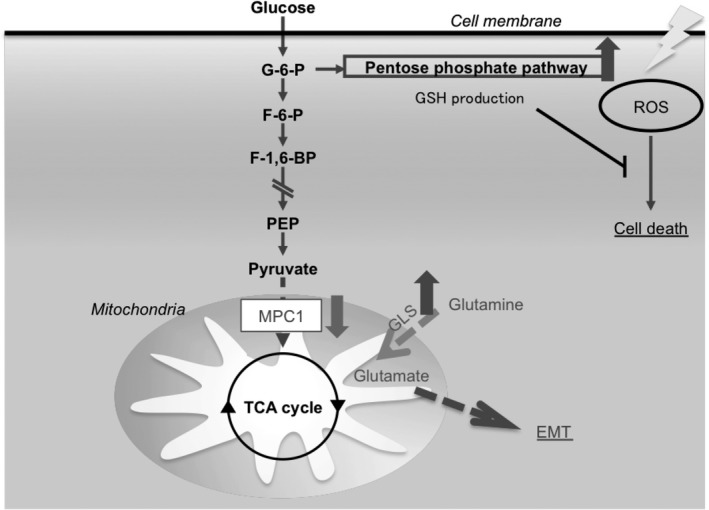
Function of low mitochondrial pyruvate carrier (MPC) expression in epithelial‐mesenchymal transition (EMT) and radioresistance. GLS, glutaminase; GSH, glutathione; PEP, pentose phosphate pathway; ROS, reactive oxygen species; TCA, tricarboxylic acid

Epithelial‐mesenchymal transition, which depends on decreased MPC expression, is thought to be due to the compensatory consumption change to other energy substrates because of a decrease in pyruvate uptake (ie, EMT is thought to be induced as a result of the metabolism of glutamine to glutamate by GLS and enhanced uptake into the mitochondria) (Figure 6). It is already known that when MPC is inhibited using UK‐5099, glutamine is metabolized by GLS and eventually enters the TCA cycle to compensate for ATP production.^9^ In contrast, it has been reported that ovarian cancer cells treat various substances, such as glutamine, fatty acids, and amino acids, as energy sources in a glucose‐deficient environment.^13^ In this study, EMT‐like changes were observed in MIA PaCa‐2 and HT29 cells, but EMT may not be induced depending on the usage trend of cell energy substrates. Regarding EMT induction, Colvin et al^10^ reported that D‐2HG, a metabolite of glutamine, is involved in the transcriptional regulation of EMT and that the amount of D‐2HG does not depend on IDH1/2 mutation. There is a possibility of a phenomenon of the same route. However, we have no evidence for involvement of D‐2HG pathway. We must further examine the type of mechanism of EMT induction observed at present.

Acquired radioresistance in MPC1 inhibitory examinations has only recently been recognized as a phenomenon, and its detailed mechanism has not yet been studied. The most likely hypothesis is the involvement of PPP. PPP is said to be the main production pathway for GSH, a radical scavenger that treats reactive oxygen species caused by ionizing radiation. It has already been reported that flux increase to PPP as a result of the suppression of function by inhibitors of MPC is involved in radioresistance.^3^ In our MPC suppression experiment, it was inferred that MPC itself does not relate to radioresistance, but flux increase to the upstream pathway relates to acquired radioresistance. PPP is also known as a major biosynthetic pathway for nucleic acids. According to known reports, the flux increase to PPP is said to be involved in gemcitabine resistance, which is a nucleic acid antagonist. It is also conceivable that a decrease in MPC1 may result in gemcitabine resistance.^4^

The acquisition of radioresistance by functional suppression using UK‐5099 and EMT by MPC1 knockdown has been reported in existing studies.^7^, ^12^ However, in the present study, MPC2 knockdown did not consistently result in EMT and radioresistance, and it was the first time that MPC1 expression alteration only was associated with EMT and radioresistance. Although MPC forms a complex of MPC1 and MPC2, there are many reports on the difference in roles of each subtype, but a consensus has not yet been determined.^6^, ^14^ Our results suggest different functions of the MPC subtype from different aspects. In contrast, it is unclear what type of mechanism causes the difference, and further examination is necessary. As a hypothesis, these results are consistent with the result of the inhibition of pyruvate transport between the cytoplasm and the mitochondria. Therefore, MPC1 and MPC2 do not have an entirely different unknown role. Reprogramming of glucose metabolism in cells by changing the existing ratio of MPC subtypes seems likely. The possibility that the proportion of each subtype in the MPC complex induces a difference in the amount of glucose into the mitochondria is an interesting point, and it is a topic for future studies.^15^

The present study has shown that reduced MPC1 expression is involved in EMT in pancreatic cancer and CRC cell lines. Such changes have not been observed in MPC2. The tendency for MPC1 expression in carcinomas to be lower in comparison to normal cells promotes EMT through glutamine metabolism. This is the first report to suggest a mechanism of new tumor metastasis and development.

Moreover, in low MPC1 expression, radioresistance is induced, which suggests that it is important to understand MPC and cancer metabolism as novel targets for cancer therapy.

## CONFLICTS OF INTEREST

Institutional endowments were received by MM, YD, and HI from Taiho Pharmaceutical Co. Ltd, Unitech Co. Ltd (Chiba, Japan), IDEA Consultants Inc. (Tokyo, Japan), and Kinshu‐kai Medical Corporation (Osaka, Japan), and by MM, YD, TS from Chugai Co., Ltd, Yakult Honsha Co. Ltd and Merck Co. Ltd. Sponsors had no role in the study design or performance, data collection, management, and interpretation, or article preparation and approval.
