# Supplementary figures and images for: Mitochondrial pyruvate carrier 1 expression controls cancer epithelial‐mesenchymal transition and radioresistance
Source: Cancer Sci. 2019 Apr 4;110(4):1331–9. doi: 10.1111/cas.13980 (PMC6447954; doi:10.1111/cas.13980)

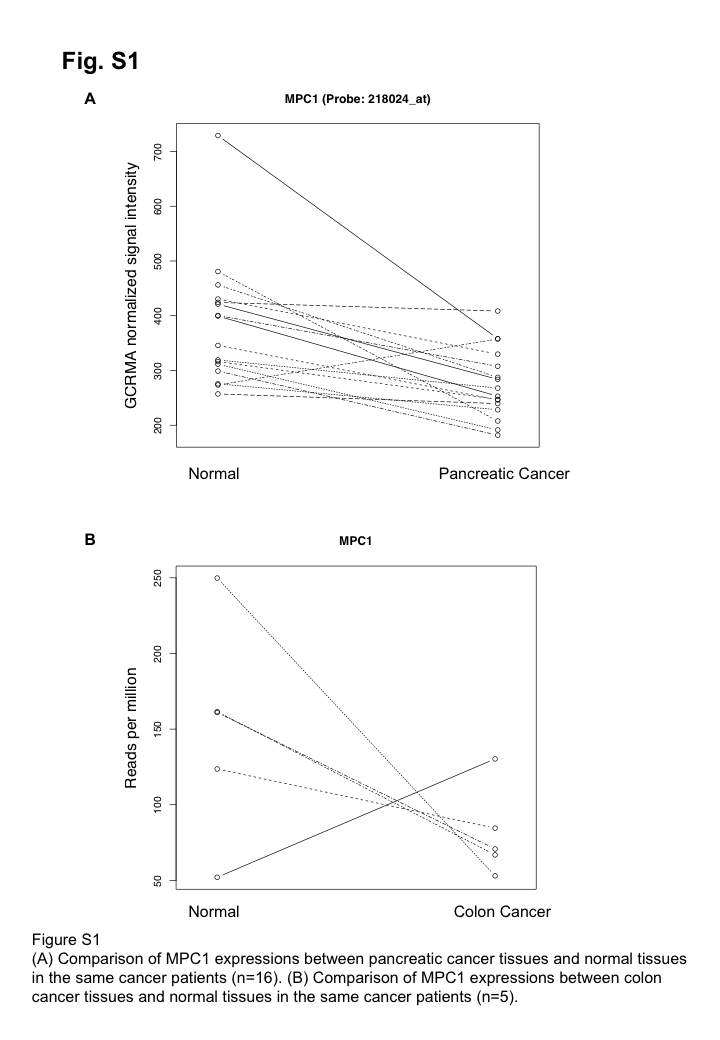

Supplement: Supplementary file 1 [file CAS-110-1331-s001.tiff]

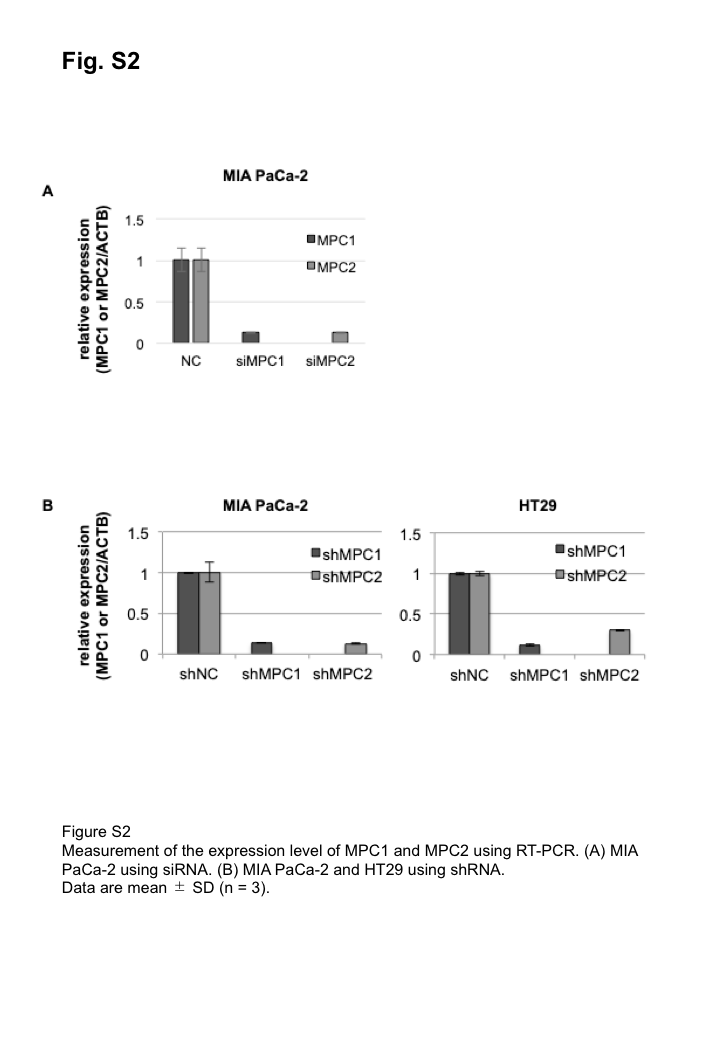

Supplement: Supplementary file 2 [file CAS-110-1331-s002.tiff]

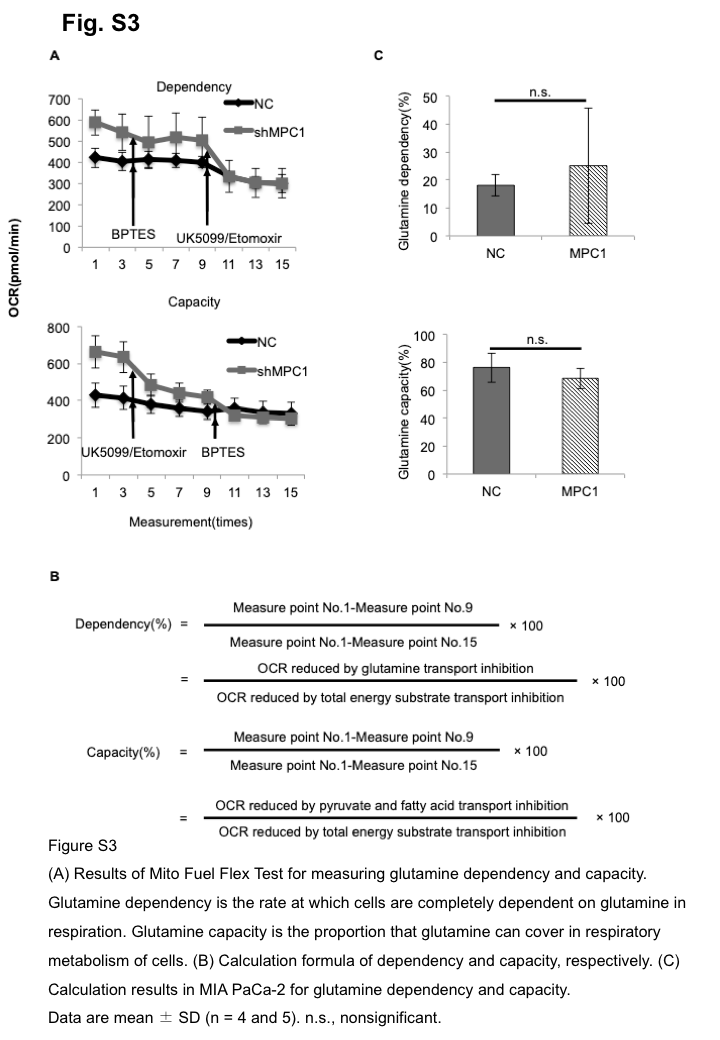

Supplement: Supplementary file 3 [file CAS-110-1331-s003.tiff]

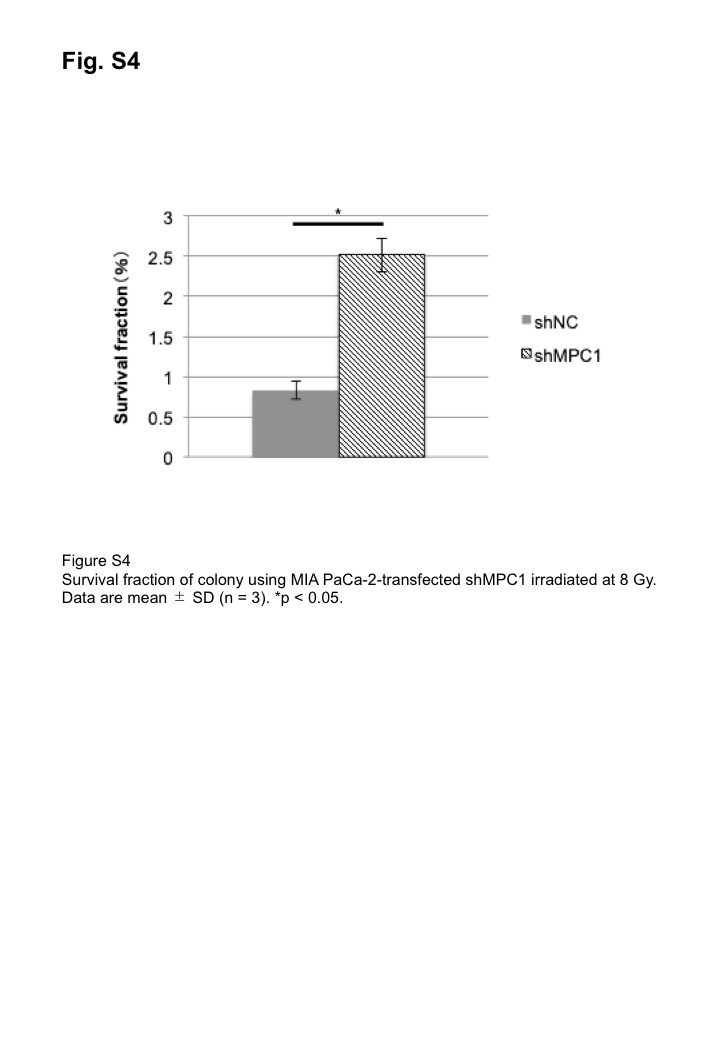

Supplement: Supplementary file 4 [file CAS-110-1331-s004.tiff]
